# Working and training through COVID-19 - completion of UK dental foundation training portfolios: a two-cohort study

**DOI:** 10.1038/s41415-023-5798-5

**Published:** 2023-05-12

**Authors:** Shyam Karia, Julian R. Joseph, Melanie L. Simms, Philip A. Atkin

**Affiliations:** 492803532249923422820 University Dental Hospital and School, Cardiff, United Kingdom; 800181315255386610734 Community Dental Officer, Cardiff and Vale University Health Board, United Kingdom

## Abstract

**Introduction** In March 2020, a cohort of dental foundation trainees (DFTs) were affected by the start of the COVID-19 pandemic. Then, in September 2020, a second cohort of DFTs began their training, with COVID-19 still affecting provision of primary dental care.

**Aims** To study the effects of COVID-19 on these two cohorts by surveying Wales' dental core trainees (DCTs) who had been undertaking dental foundation training (DFTg) in 2019/20 and 2020/21.

**Materials and methods** Following ethical approval, we conducted two online surveys for the 2019/20 and 2020/21 DFTs' cohorts. We compared and contrasted their reported completion of various DFTg curriculum components and any additional skills derived from redeployment.

**Results** A response rate of 52% was achieved for both surveys. All participants successfully completed DFTg; although, some small differences were noted between the cohorts and their ability to fulfil all their portfolio requirements.

**Discussion** Despite the effects of COVID-19, all DFTs were able to demonstrate completion of curriculum elements. The redeployment of three DFTs enhanced their learning. This was comparable to reports from other DFTs who were redeployed in the pandemic.

**Conclusions** All DCTs surveyed from both cohorts successfully completed their DFTg portfolios. In some cases, additional skills were developed, which in the absence of the pandemic, may not have been the case.

## Key points

- Two cohorts of dental foundation trainees (DFTs) were most affected by the COVID-19 pandemic, and we sought to find out more about their experiences of training during this period.
- Despite the restrictions caused by the pandemic on the ability of dental practices to open and provide care, DFTs managed to successfully complete their training.
- Some redeployed DFTs experienced patient-care scenarios well outside of the dental foundation training curriculum, and felt they benefited from these non-core training experiences.

## Introduction

The World Health Organisation declared COVID-19 a pandemic on 12 March 2020^1^ and soon after, the NHS in Wales and England advised postponement of all elective procedures, including dentistry.^2^^,^^3^ Dental school/hospitals and dental practices across the UK were all affected. Dental hospitals immediately became urgent dental care centres, and many dental staff were redeployed into acute medical service support.^4^ Recent graduates - dental foundation trainees (DFTs) - who had been in general dental practice-based posts from September 2019 were also affected, with some being redeployed into the newly-created COVID-19-specific Nightingale hospitals.^5^ As the pandemic progressed along the academic year, newly graduated dentists began their dental foundation training (DFTg) in September 2020, with significant restrictions still affecting the provision of services in general dental practice. The UK General Dental Council put out a statement in March 2020 describing what they hoped would happen to final year dental students and recent graduates (DFTs) as the pandemic unfolded.^6^

Currently, DFTg follows a structured curriculum, that was published in 2015 by the UK Committee of Postgraduate Dental Deans and Directors (COPDEND).^7^ This *Dental foundation training curriculum* implements learning outcomes and an electronic training portfolio (e-portfolio) to allow DFTs to log their activities and reflections.^8^ The use of portfolios to support learning is widespread in dental education. They are encouraged in the Association for Dental Education in Europe's 'Graduating European dentist' curriculum and used for UK dental core training and speciality registrar training.^9^^,^^10^^,^^11^ At 6 and 12 months, DFTs will have a review of competence progression (RCP) recorded in their portfolio. This documents their progress through four main training domains, as well as the completion of workplace-based assessments (WBAs) and clinical competencies.

On completion of DFTg, a number of dentists will progress into dental core trainee (DCT) posts. These salaried posts are typically located in dental community clinics and dental teaching or district general hospitals, where there are opportunities to gain further experience in several dental specialties or in maxillofacial surgery, while also taking part in formal study days.

The aim of this study was to ascertain how the two cohorts of DFTs who were most affected by the COVID-19 pandemic felt they were able to complete the learning outcomes within the DFTg curriculum and linked e-portfolio, and if COVID-19 offered any opportunities to develop additional knowledge or skills.

## Materials and methods

Inclusion criteria for this study was any DCT working in the Wales Deanery who had completed their UK DFTg year during the COVID-19 pandemic (2019/20 and 2020/21).

Following ethical approval (Cardiff University Dental School Research and Ethics Committee [DSREC] Refs 2032a and 2116a), we conducted two studies (in 2021 and 2022) in which we asked DCTs working in Wales from September 2020 to August 2021, and September 2021 to August 2022, about their experiences of DFTg. Participation in the study was entirely voluntary and those that chose to begin completion of the online surveys having read the participant information sheet were able to abandon at any time. No identifying participant data were collected. Consent to participate was implied by completion of the survey.

The links to online surveys were distributed to the two cohorts of Wales DCTs by the Wales Dental Postgraduate Deanery, Health Education and Improvement Wales (HEIW).^12^^,^^13^ The surveys collected basic anonymous demographic data about the DCTs, including where in the UK they had completed their DFTg programme. It also assessed whether they had been able to attend study days during their training, if they had been able to complete the WBAs, and other elements described in the DFTg curriculum. These included the four principal domains of: clinical; communication; professionalism; and management and leadership. The online survey questions used Likert scale answer options for ease and speed of completion, and free-text boxes were offered to allow additional comments or clarifications to be made where necessary.^14^

## Results

### Survey participants and dental foundation training deanery locations

Both studies achieved a response rate of 52% (n = 17 in each study). The results indicated that the majority of respondents from both cohorts completed their DFTg within HEIW/Wales Deanery. This amounted to 52.9% for the 2019/20 cohort and 46% for the 2020/21 cohort. Other respondents completed their DFTg in Health Education England (HEE) deaneries: HEE West Midlands; HEE East Midlands; HEE London; HEE South West; and HEE Kent, Surrey and Sussex. No participants had completed DFTg in Scotland or Northern Ireland, or an equivalent training outside of the UK.

### Completion of COPDEND dental foundation training curriculum domains

Within the 2019/20 cohort, 82% of respondents agreed/strongly agreed that the 'clinical' curriculum domain was satisfactorily completed and 94% agreed/strongly agreed that the 'communication' domain was satisfactorily completed. This improved for the 2020/21 cohort - 100% agreed/strongly agreed that both the 'clinical' and 'communication' domains were satisfactorily completed.

**Fig. 1:**
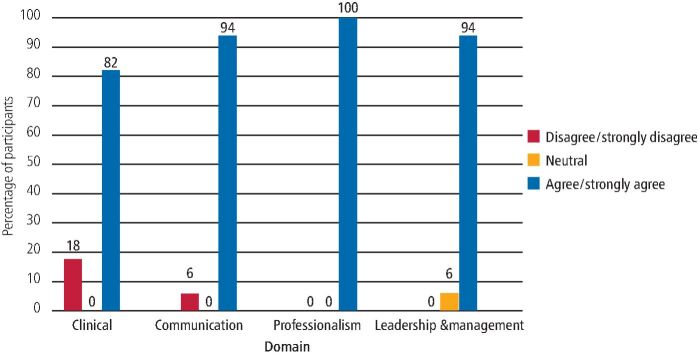
Completion of curriculum domains - 2019/20 DFTs cohort

**Fig. 2:**
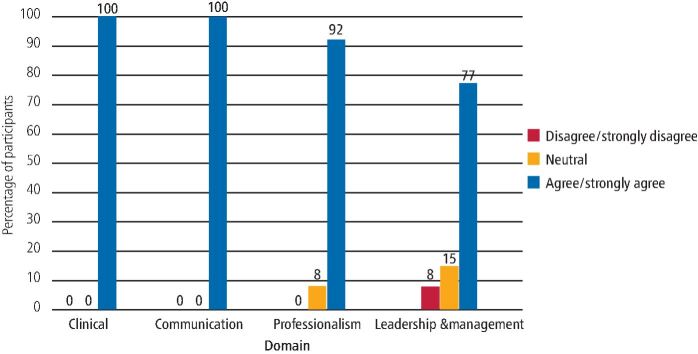
Completion of curriculum domains - 2020/21 DFTs cohort

Of the 2019/20 cohort, 100% agreed/strongly agreed that the 'professionalism' domain was satisfactorily completed, compared to 92% of the 2020/21 cohort. Likewise, for the 'management and leadership' domain, 94% of the 2019/20 cohort reported satisfactory completion compared to 77% from the 2020/21 cohort. Figure 1 and Figure 2 show the completion of the curriculum domains for DFTg in the 2019/20 and 2020/21 cohorts, respectively.

### Completion of workplace-based assessment

The completion of a number of WBAs was assessed, including multisource feedback (MSF), patient satisfaction questionnaires (PSQ), dental evaluation performance tools (ADEPT [A Dental Evaluation of Performance Tool]) and case-based discussions (CBD). All participants from both cohorts reported fully meeting the predefined requirements for MSF and PSQ.

With regards to the completion of ADEPTs and CBDs, all participants within the 2020/21 cohort met the required number of each by the final RCP deadline. In comparison, 17.9% and 5.9% of participants from the 2019/20 cohort expressed difficulties meeting the required totals of ADEPTs and CBDs, respectively.

### Tutorials and study days

Tutorials are one aspect where significant differences were noted between the cohorts. In the 2019/20 cohort, 76.5% of participants reported that their tutorials were being rearranged for online delivery, in contrast to 23% from the following year. Moreover, only 64.7% described receiving a minimum of 31 tutorials in 2019/20, compared to 84% in 2020/21.

Less profound differences were reported in regard to DFTg study days. Most participants from both cohorts (82.4% from 2019/20 and 92% from 2020/21) indicated they received 21 or more study days. Although both cohorts reported that a large proportion of their study days were rearranged for online delivery, only 8% of the 2020/21 cohort reported receiving 15 or more face-to-face study days, compared with 88.2% from the 2019/20 cohort.

### Clinical activity records

**Fig. 3:**
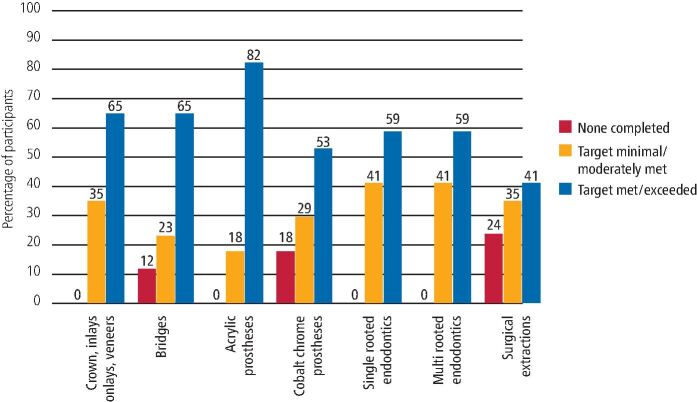
Completion of clinical activity - 2019/20 DFTs cohort

**Fig. 4:**
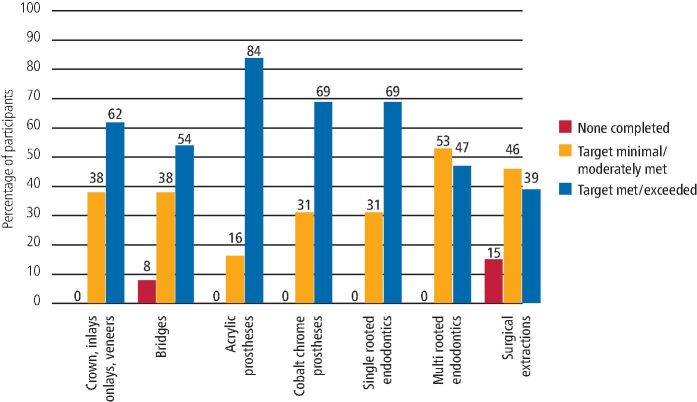
Completion of clinical activity - 2020/21 DFTs cohort

**Fig. 5:**
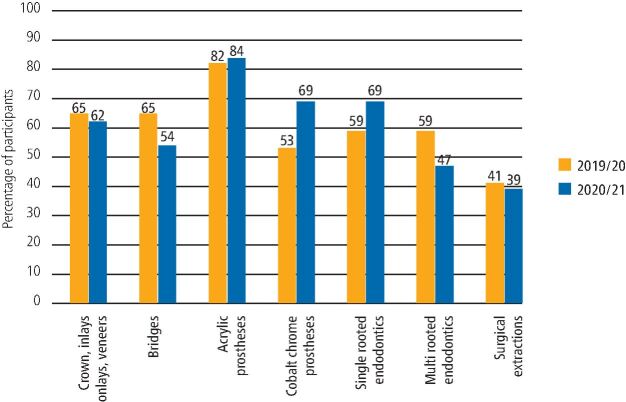
Comparison showing the percentage of participants meeting or exceeding their clinical quotas

A comprehensive selection of clinical activities was assessed to determine how confident DFTs felt about fulfilling their clinical criteria. Figure 3 and Figure 4 show the completion of clinical activity records by the 2019/20 and 2020/21 cohorts. Figure 5 directly compares both cohorts, demonstrating the percentage of participants who reported to meet or exceed their clinical quotas.

### Crowns, inlays, onlays and veneers

All DFTs completed at least one crown, inlay, onlay or veneer. Moreover, 65% of participants from the 2019/20 cohort reported meeting or exceeding their quota and a similar figure of 62% was obtained from the 2020/21 cohort. Additionally, 35% of participants from 2019/20 reported fulfilling their quota to a 'minor or moderate extent', compared to 38% from the 2020/21 cohort.

### Bridges

In total, 12% of participants from the 2019/20 cohort and 8% from 2020/21 reported not having completed a bridge during their DFTg year, and 65% of participants from the 2019/20 cohort met or exceeded their quota compared to 54% from 2020/21.

### Acrylic prosthesis

In total, 82% of DFTs from the 2019/20 cohort and 84% from the 2020/21 cohort met or exceeded their quota in completing acrylic prostheses. All other participants fulfilled their quota to a 'moderate extent', except one participant from 2019/20, who reported only meeting their quota to a 'minor extent'.

### Cobalt chrome prosthesis

All participants from the 2020/21 cohort completed at least one cobalt chrome prosthesis during their DFTg year, compared to 82% from the 2019/20 cohort. Moreover, 53% of the 2020/21 cohort met or exceeded their required targets compared to 69% from 2020/21.

### Endodontic cases

All participants completed at least one single-rooted and one multi-rooted endodontic treatment. Within the 2019/20 cohort, 59% reported meeting or exceeding their set quota for both single-rooted and multi-rooted endodontic cases. Likewise, for the 2020/21 cohort, 69% reported meeting or exceeding the set quota for single-rooted cases and 47% for multi-rooted cases.

### Surgical extractions

In both cohorts, out of all clinical procedures, participants reported having the least clinical exposure in surgical extractions. Here, 24% of the 2019/20 cohort and 15% of the 2020/21 cohort reported completing zero surgical extractions, and 41% from the 2019/20 cohort met or exceeded their set quota, compared with a figure of 39% from 2020/21.

## Discussion

McNulty (2008) compared paper-based and online surveys, noting response rates of between 20-47% for online surveys,^15^ while Archer (2008)^16^ concluded that 'impact evaluation' surveys (such as this study) produced a mean response rate of 51.4%. Thus, our response rate of 52% is reasonable and the results can be generalised to the wider population of DCTs and their experiences of DFTg in COVID-19. In an attempt to improve the response rate, we sent reminder emails at weeks two and three which included the survey link (which was open until week four). Reminder emails have been shown to improve response rates.^17^

For this survey of Wales Deanery DCTs, around half had completed their DFTg within Wales. The remainder were from different deaneries in England. Both cohorts will have been applying for DCT posts during the peak of COVID-19 and perhaps this influenced their decision to stay working in the local vicinity. Travel across the UK was discouraged during COVID-19, so the practicalities of moving around the UK and having to find accommodation during these restrictions may have influenced this outcome. It may also explain the lack of Wales DCTs who completed DFTg in Scotland and Northern Ireland.

In relation to completion of portfolio curriculum domains, the majority of both cohorts felt they were successful in all four areas. The 2019/20 cohort - for whom the second part of their DFTg programme was disrupted by COVID-19 - reported slightly lower percentages for the 'clinical' and 'communication' domains than the 2020/21 cohort. This later cohort entered into DFTg when the disruption of training had perhaps been ameliorated by the deaneries and local scheme organisers, who had put in place online or remote learning opportunities that took some time to organise for the first cohort. This pattern was reversed for the 'professionalism' and 'management and leadership' domains, but only slightly, with 77% being the lowest proportion to report completion of the 'management and leadership' domain.

Both cohorts completed 100% of their WBAs, although the earlier cohort reported difficulty in meeting the ADEPT and CBD portfolio outcomes. Again, this may be because of the greater disruption to their study days and dental practice opportunities, given the sudden onset of COVID-19 restrictions for the latter half of their DFTg year. Unsurprisingly, both cohorts reported disruption to their trainer tutorials and group study days, with a conversion from face-to-face meetings to online sessions appropriate for reducing travel and mixing with colleagues as part of the national effort to reduce COVID-19 transmission in the community.

As each deanery is responsible for setting their own clinical quotas, there is slight variability between the clinical targets DFTs are expected to achieve nationally. These targets are not always specified online and are generally communicated directly to DFTs. Due to this, it was challenging to accurately deduce how clinical quotas changed during the course of COVID-19, but from available data, we noted that clinical targets stayed consistent and appeared fair.

In relation to clinical activity reported by both cohorts, variable numbers of DFTs were able to meet or exceed the requirements for restorative dentistry procedures listed in the e-portfolio. Reviewing Figure 3 and Figure 4 for clinical activity (mostly restorative procedures), the 2019/20 cohort may have struggled more to meet their targets. This might be because as the DFTg year progresses, newly qualified dentists become more confident and competent to deliver more of the complex restorative treatments, such as crowns, inlays, veneers and bridges, and this crucial second part of the DFTg year was suddenly cut short for the 2019/20 cohort. In comparison with the 2020/21 cohort, due to existing knowledge of reduced clinical activity because of aerosol generating procedure (AGP) restrictions, and patient numbers and flow through the practice, they were better placed to plan and pace their progress through the year.

In addition to surveying the DFTg e-portfolio elements, we asked both cohorts if they felt that they had gained any additional skills as a result of any changed activities or opportunities created by the disruption to training caused by COVID-19. Both cohorts felt that they had 'an improved ability to cope with stressful situations' (59% of 2019/20; 85% of 2020/21), 'improved teamworking skills' (53% of 2019/20; 62% of 2020/21), 'improved communication skills (47% of 2019/20; 69% of 2020/21) and 'improved ability to cope with dental emergencies' (53% 2019/20; 46% of 2020/21).

In relation to redeployment, only one DFT was redeployed in 2019/20, to answer NHS 111 calls (non-emergency medical advice), and in the 2020/21 cohort, two DFTs were redeployed; one to a COVID-19 testing facility, and another to an urgent dental care centre.

There are few published accounts of DFT experiences in the first year of the COVID-19 pandemic in the UK literature. Johnson *et al.* (2020)^5^ described the experience of four DFTs, all of whom were 2019 dental graduates and were re-deployed to a Nightingale hospital at the beginning of COVID-19. They linked their experiences to their DFTg portfolio, and statements from their paper include:

- 'Working at Nightingale was different from our initial expectations of a dental foundation training year. However, this has been a learning opportunity that links back to our dental foundation curriculum. For example, understanding the dynamics of multi-professional working, reflection and recognition of educational opportunities. We can appreciate the positive aspects of dentistry, in particular, continuity of care, developing rapport and seeing patients on a regular basis'
- 'The devotion and care which our colleagues gave to the patients truly inspired us. This reinforced why we pursued a career in healthcare and highlighted the need for self-reflection and continual development'
- 'Upon returning to practice, we will bring the lessons we learned in communication, how to implement a positive work culture and the importance of common purpose with a wider perspective of health care outside of dentistry'.

In addition, Jalota (2021), a dental graduate of 2020, describes her experience of beginning DFTg in a dental practice within HEE West Midlands when COVID-19 was already established in the community. Along with fellow DFTs, she was also deployed to a COVID-19 testing centre.^18^Jalota reported:

- 'Fallow times were being positively utilised in many ways, such as by having tutorials with my trainer, practising on phantom head teeth and learning how to use the clinical computer software appropriately'
- 'Deployment also had its advantages. Ultimately, it brought everyone on my scheme closer, as we were spending a lot of time together'
- 'Helping with COVID-19 swab tests made me feel as though I was part of something much bigger and I liked meeting people from a wide range of communities'.

## Conclusion

We believe this is the first study to describe the experiences of two cohorts of DCTs, the fulfilment of their DFTg e-portfolios during the first two years of the COVID-19 pandemic, and their experiences of redeployment with links to the e-portfolio learning outcomes. Despite the immediate chaos caused by the sudden shutdown of DFTg practices in March 2020 - and the development of COVID-19-secure dental treatments, AGPs requiring enhanced personal protective equipment/fallow times, and the disruption to the planned training events of DFTg - the surveyed DCTs reported completion of their DFTg e-portfolios and successfully progressed out from DFTg. Some DFTs gained additional knowledge and insight into the wider working of the NHS - which they may not have done were it not for the pandemic.
